# Association between appendicular lean mass and chronic obstructive pulmonary disease: epidemiological cross-sectional study and bidirectional Mendelian randomization analysis

**DOI:** 10.3389/fnut.2023.1159949

**Published:** 2023-06-29

**Authors:** Chengjie Fu, Hongchang Yang

**Affiliations:** Department of Physical Education, Hohai University, Nanjing, Jiangsu, China

**Keywords:** chronic obstructive pulmonary disease, appendicular lean mass, cross-sectional study, NHANES, Mendelian randomization, epidemiological study

## Abstract

**Background:**

The association of BMI with COPD, and sarcopenia in COPD have been both confirmed by several studies, but research on the relationship and causality of body lean mass and the risk of chronic obstructive pulmonary disease (COPD) remains to be discovered. The purpose of this study was to explore the association between lean mass and COPD risk as well as to further examine the causal relationship in the findings.

**Methods:**

Three thousand four hundred fifty-nine participants from NHANES 2013–2018 were included in the epidemiological cross-sectional study to assess the association between relative lean mass and COPD by restricted spline analysis (RCS) and weighted multiple logistic regression. Furthermore, to verify the causality between lean mass and COPD, a two-sample Mendelian randomization (MR) with inverse variance weighting (IVW) method was used to analyze GWAS data from European ancestry. Genetic data from the United Kindom Biobank for appendicular lean mass (450,243 cases) and lung function (FEV_1_/FVC) (400,102 cases) together with the FinnGen platform for COPD (6,915 cases and 186,723 controls) were used for MR.

**Results:**

Weighted multiple logistic regression showed a significant correlation between relative appendicular lean mass and COPD after adjusting for confounders (OR = 0.985, 95% CI: 0.975–0.995). Compared to the lower mass (155.3–254.7) g/kg, the high mass (317.0–408.5) g/kg of appendicular lean apparently decreases the risk of COPD (OR = 0.214, 95% CI: 0.060–0.767). Besides, in the analysis of MR, there was a forward causality between appendicular lean mass and COPD (IVW: OR = 0.803; 95%CI: 0.680–0.949; *p* = 0.01), with a weak trend of causality to lung function.

**Conclusion:**

Our study not only found an inverse association between appendicular lean mass and COPD but also supported a unidirectional causality. This provided possible evidence for further identification of people at risk for COPD and prevention of COPD based on limb muscle exercise and nutritional supplementation to maintain skeletal muscle mass.

## 1. Introduction

Chronic obstructive pulmonary disease (COPD), a leading cause of chronic morbidity and mortality, is a significant global public health issue, and a heavy disease burden worldwide (1, 2). It is a common and preventable disease characterized by persistent progressive airflow limitation and is associated with an increased inflammatory response of the airways and lungs to toxic particles or gasses (3). By 2030, the number of patients diagnosed with COPD is expected to increase by 155% and the rate of hospitalizations associated with COPD is predicted to increase by 210% (4). Therefore, the high burden and prevalence of COPD have prompted a greater emphasis on early prevention and identification.

Body mass index (BMI) is recognized to be associated with COPD (5), and low BMI is an independent risk factor for death in COPD patients (6). However, previous studies based on BMI had limitations, in which a reduction in skeletal muscle mass, usually occurring in the early stages of COPD, was the main cause of weight loss in COPD patients, rather than a reduction in BMI (7). What’s more, Behrens et al. showed that underweight BMI adjusted for waist circumference, which may precisely represent an indirect marker of low muscularity, was positively associated with COPD, especially in older adults (8). These were somewhat suggestive of a possible negative association between lean mass and the occurrence of COPD, which was lacking in previous studies, so we validated this association hypothesis currently through an epidemiological cross-sectional study.

Additionally, progressive loss of muscle mass and function is significant systemic effects characteristics of COPD (9, 10). Therefore, the association between lean mass and COPD derived from observational cross-sectional studies alone cannot avoid reverse causality and numerous confounding factors to determine reliable conclusions. In this case, Mendelian randomization studies can provide evidence to further explore causal associations. Mendelian randomization (MR) can be considered similar to randomized controlled trials (11) and draws on genetic variation as an instrumental variable (IV) to infer a causal relationship between exposure and outcome (12). Due to the random assortment of genes from parents to offspring that happens during gamete formation and conception, genetic variants are attractive as candidate IVs that effectively avoid reversing causality bias and confounding bias of cross-sectional study (13). Furthermore, since the consequences of genetic variation are more prolonged than interventions, the real causal effects calculated by MR may be more strong and more reliable (14). Likewise in previous studies, there were MR studies that studied the relationship between lean mass and diabetes (15), coronary artery disease (16), and osteoporosis (17).

Thus, this study aimed to conduct a preliminary cross-sectional study of the association between whole-body total lean mass and appendicular lean mass to COPD risk using the wide database of the National Health and Nutrition Examination Survey (NHANES), followed by a further bidirectional MR study to assess causal relationships at the level of genetic variation. This provided possible evidence for identifying people at risk for COPD and maintaining lean mass for COPD prevention.

## 2. Materials and methods

### 2.1. Epidemiological cross-sectional study design and data source

**Figure 1:**
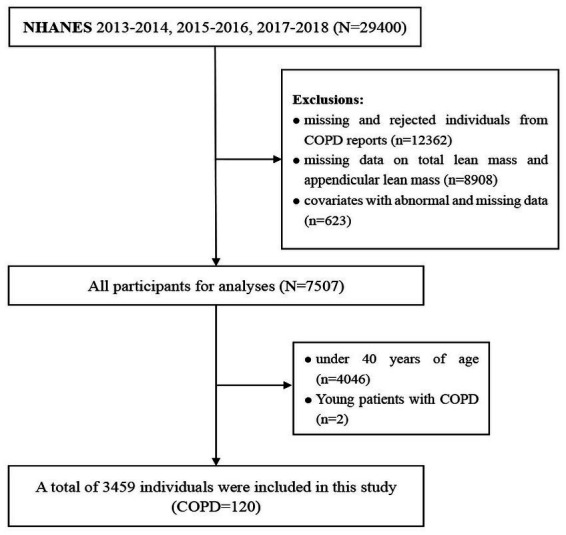
Flow chart of participants screening in NHANES 2013–2018.

NHANES is a large cross-sectional population survey, administered by the Centers for Disease Control and Prevention (CDC) that consists primarily of interviews and physical examinations. The survey was approved by the Institutional Review Board of the National Center for Health Statistics (NCHS), which obtained informed consent from all participants (18). Data from three cycles in the database including 2013–2014, 2015–2016, and 2017–2018 were integrated for analysis (*n* = 29,400). Then, we excluded missing and rejected individuals from COPD reports (*n* = 12,362), and a total of 120 adults between the ages of 40 and 60 with COPD were selected for the final analysis. Individuals, under 40 years of age (*n* = 4,046), missing data on total lean mass and appendicular lean mass (*n* = 8,908), and covariates with abnormal and missing data (*n* = 623) were excluded (Figure 1). Finally, a total of 3,459 individuals were included in the final logistic regression analysis. By utilizing a simulation algorithm that adjusts propensity weights, the effect of biased sampling was lessened. Detailed data is available on the NHANES website.^1^

### 2.2. All variables included in the cross-sectional analysis

Individual disease conditions were obtained and confirmed through interviews, with the medical condition section of the questionnaire providing self-reported personal data on various health conditions and medical histories, and interviews were conducted at home by trained interviewers using the Computer Assisted Personal Interviewing (CAPI) system. Therefore, COPD was confirmed based on the Medical Conditions Questionnaire (MCQ), in which individuals were asked “Has a doctor or other health professional ever told you had COPD?”

Total lean mass (g) and appendicular lean mass (g) measured in all eligible participants were extracted during the survey period (2013–2018). Appendicular lean body mass (g), a recognized proxy for skeletal muscle mass, was determined by summing the lean mass (excluding bone mineral content) of the upper and lower extremities measured by dual-energy X-ray absorptiometry (DEXA) (19, 20). Most studies have used bioimpedance-based surrogates of lean mass and limited studies have been performed using DEXA to assess body composition (21), but DEXA, available for diagnosis of sarcopenia (22), is the preferred method of body composition measurement for better muscle mass assessment (23). All variables measured by DEXA were shown relative to body mass (g/kg BM) to explain the effect of body mass on differences in these outcomes. Participants over 59 years of age were not qualified for DEXA measurement (24).

The following variables were used as covariates according to previous studies: gender, age, race, education level, the ratio of family income to poverty (PIR), body mass index (BMI), smoking status, alcohol intake, sedentary time, physical activity level, hypertension, and diabetes status. Physical activity was obtained from the Global Physical Activity Questionnaire (GPAQ) collected during interviews (25, 26). we calculated the value of PA quantitatively from the following formula: PA (MET-min/W) = MET× Weekly Frequency× Duration of PA (27, 28). In addition, participants were considered smokers if they had smoked ≥100 cigarettes in their lifetime, and smoking was categorized based on self-reported: Smoking status (never, former, current) (29). Self-reported frequency of alcohol intake was also classified (never/special occasion, 1–3 times per month, 1–4 times per week, daily/almost daily). Overweight was defined as having a BMI between >25 and ≤ 30 kg/m^2^ while obese was defined as having a BMI > 30 kg/m^2^. Social economic status was classified using PIR, which was categorized as low (PIR ⩽ 1.3), middle (PIR > 1.3 to ⩽3.5), or high (PIR > 3.5) status.

### 2.3. Sources of bidirectional MR

The United Kingdom Biobank is a major biomedical database and research resource with in-depth genetic and health information from over half a million United Kingdom participants.^2^ Appendicular lean mass was quantified by summing the fat-free mass of 450,243 participants of the UK Biobank cohort (ID: ebi-a-GCST90000025), adjusted for appendicular fat mass, age, and other covariates (30). Appendicular lean mass is important as a measure of muscle mass in older adults and allows the identification of clinically significant frailty (31).

GWAS data for Lung function (FEV_1_/FVC) closely associated with COPD diagnosis included 400,102 individuals from the United Kingdom Biobank and the SpiroMeta database (ID: ebi-a-GCST007431) (32). In particular, GWAS data for COPD were obtained from FinnGen,^3^ a large genomic research project developed in Finland, and the COPD GWAS from FinnGen included 6,915 cases and 186,723 controls (ID: finn-b-J10_COPD). The populations included in our analysis were mainly of European origin, which is publicly available and can be searched at the IEU OpenGWAS database according to ID.^4^

### 2.4. Statistical analysis of the cross-sectional study

In cross-sectional statistical analysis, baseline characteristics of all participants were described by average (continuous variables; expressed as mean ± standard deviation (SD)) or proportion (categorical variables; expressed as N (%)). Six-year sample weights (2013–2018) were generated by combining the two-year sample weights for each survey cycle, as described in the NHANES guidebook. In addition, the data distribution was adjusted according to the Mobile Examination Center (MEC) sample weights to handle the over-sampling of the data itself. Since the percentage of missing data was less than 10%, the mean or plural of the sample was used directly to fill in the missing values.

To explore the relationship between lean mass and COPD risk, logistic regression and a restricted cubic spline model (RCS) were used to analyze and fit the graphs. Finally, we selected 3 knots in the main spline curve of COPD to achieve a better curve fit. Subsequently, we further applied quintiles to combine successive lean mass into three categories to arrive at a reasonable effect to assess the OR value for three different groups, which was performed using a dichotomous logistic regression model. We used the lean mass level category closest to OR = 1 in the fitted curve as a reference so that we could roughly distinguish between high and low muscle level groups, which were eventually combined into three groups based on quintiles. All the above model constructions were adjusted several times according to the confounding variables. Ultimately, we performed weighted multiple logistic regression by adjusting for different confounding variables to construct three models to calculate the odds ratio (OR), value of p, and 95% confidence interval (CI) between lean mass and COPD risk.

### 2.5. Bidirectional mendelian randomization analysis

**Figure 2:**
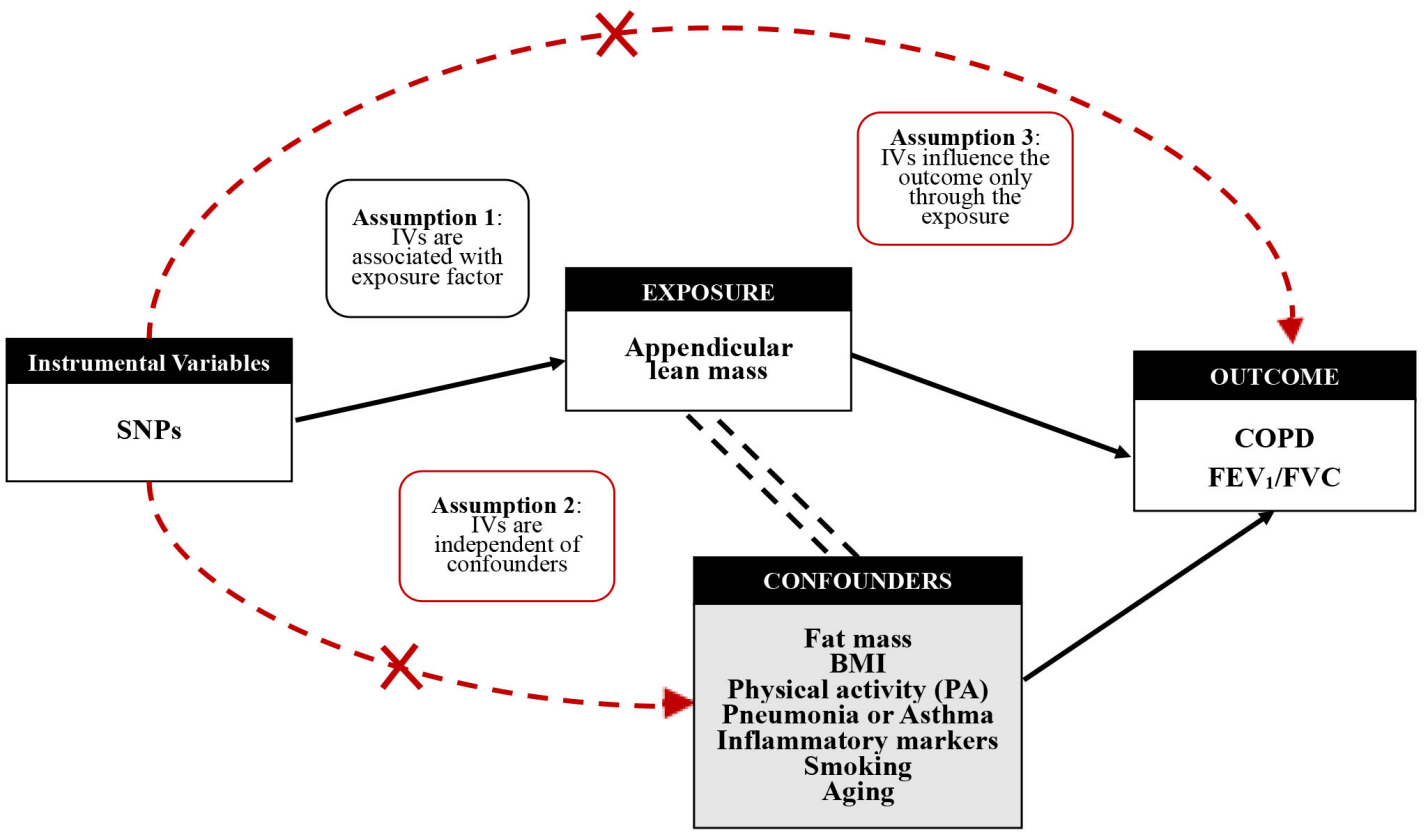
Mendelian randomization study design and genetic instrumental variables (IVs) excluding confounding factors.

Bidirectional two-sample MR analysis is a method that employs genetic instrumental variables (IV) to assess the direction of causality between exposure and outcome. Three hypotheses guided the analysis of MR (1): IV is associated with exposure factor (2), IV is independent of confounder, and (3) IV contributes to outcome through exposure factors only (Figure 2). To construct genetic instrumental variables for exposure factors, reliable SNPs (single nucleotide polymorphisms) were obtained and trimmed to remove the effect of linkage disequilibrium (LD) (*p* < 5 × 10^−8^ or *p* < 5 × 10^−10^, *r*^2^ < 0.001, kb = 10,000) (33). We chose not to use SNP proxies, coordinated the effect alleles in the exposure and outcome datasets, and excluded all SNPs with palindromes. To obtain reliable estimates, the inverse variance weighted (IVW) model, MR-Egger regression model, weighted median estimators, weighted mode, and simple mode were used to explore a causality, followed by testing for imbalance horizontal pleiotropic effects, and IVW was considered as the main analysis method (34). We focused on testing horizontal pleiotropic with the R package, and when the value of the intercept term is approaching zero and the *p*-value is more than 0.05, it means that there is no horizontal pleiotropic (35). In addition, heterogeneity effects between genetic instrumental variables were identified and considered by removing heterogeneous SNPs using the MR-PRESSO package. In the formula below, the F statistic is employed to detect a weak instrumental variable, *R*^2^ is the variance of exposure factors interpreted by the genetic instruments, and K, taken as 1, is the amount of genetic variation since the *F* value of each SNP is calculated in this paper, and N is the number of samples. The calculated F value greater than 10 indicates that weak IVs bias is unlikely and these IVs can be included in the study (36). Finally, sensitivity analysis was performed, in which SNPs were removed one by one to test the effect of each SNP, using the leave-one-out method, besides a forest plot to observe the effect of each SNP. A similar analytical procedure was repeatedly applied, swapping exposure and outcome factors to achieve a bidirectional MR study to assess reverse causality (37).

The formula for calculating *R*^2^:


$$R^{2}=2\times \left(1-MAF\right)\times MAF\times {\left(\frac{\beta }{SD}\right)}^{2}$$

The formula for calculating the F-statistic:


$$F=\frac{N-k-1}{k}\times \frac{R^{2}}{1-R^{2}}$$

All data analyzes were performed by R software (Version 4.2.2)^5^ and STATA 16 (Stata Corporation, College Station, TX, United States). *P* < 0.05 indicates statistical significance.

## 3. Results

### 3.1. Epidemiological cross-sectional study observation and analysis

The participant characteristics in the statistical analysis were obtained from NHANES 2013–2018. We found that 120 patients (3.5%) of the 3,459 participants in the study were diagnosed with COPD based on their self-report status. Table 1 provides all available characteristics of participants subdivided by COPD, with statistically significant differences between groups in weighted and unweighted data for multiple indicators. The COPD group population was significantly older than the non-COPD group, and there were major differences in gender between the two groups. In addition, the non-COPD group was more highly educated and generally better off, as evidenced by a higher PIR. In addition, we found that the mass of relative total lean and relative appendicular lean in the non-COPD group was 632.14 ± 79.74 g/kg and 274.01 ± 45.69 g/kg, respectively, while the mass of relative total lean mass and relative appendicular lean mass in the COPD group were 611.68 ± 84.00 g/kg and 251.23 ± 42.98 g/kg. In the case of these significant differences, we attempted to further explore the possible linear or nonlinear relationship between lean mass and COPD risk by employing RCS analysis.

**Table 1:** Baseline characteristics of study participants with or without COPD in the NHANES 2013–2018 (unweighted and weighted).

Variables	Non-COPD(% or mean ± SD)	COPD(% or mean ± SD)	*p*-value	*p*-value*
All participants	3,339 (96.53%)	120(3.47%)	–	–
Gender			0.009	0.003
Male	1,603 (48.01%)	43 (35.83%)		
Female	1736 (51.99%)	77 (64.17%)		
Age	49.23 ± 5.72	52.15 ± 5.11	<0.001	<0.001
Race			<0.001	0.055
Mexican American	523 (15.66%)	5 (4.17%)		
Other Hispanic	347 (10.39%)	6 (5.00%)		
Non-Hispanic White	1,161 (34.77%)	80 (66.67%)		
Non-Hispanic Black	685 (20.52%)	13 (10.83%)		
Other race	623 (18.66%)	16 (13.33%)		
Educational level			<0.001	<0.001
Less than 9th grade	245 (7.34%)	12 (10.00%)		
9-11th grade	367 (10.99%)	30 (25.00%)		
High school graduate	731 (21.89%)	37 (30.83%)		
Some college	1,042 (31.21%)	34 (28.33%)		
College graduate or above	954 (28.57%)	7 (5.83%)		
The ratio of family income to poverty			<0.001	<0.001
Low (≤ 1.3)	879 (26.33%)	79 (65.83%)		
Middle (≤ 3.5 and >1.3)	1,216 (36.42%)	31 (25.83%)		
High (>3.5)	1,244 (37.26%)	10 (8.33%)		
BMI			<0.001	<0.001
Low (<25)	840 (25.16%)	46 (38.33%)		
Middle (≤ 25 and <30)	1,159 (34.71%)	21 (17.50%)		
High (≤ 30)	1,340 (40.13%)	53 (44.17%)		
Smoking status			<0.001	<0.001
Current	714 (21.38%)	80 (66.67%)		
Former	684 (20.49%)	22 (18.33%)		
Never	1941 (58.13%)	18 (15.00%)		
Alcohol drinking			0.002	<0.001
Daily/Almost daily	250 (7.49%)	4 (3.33%)		
1–4 times per week	787 (23.57%)	14 (11.67%)		
1–3 times per month	823 (24.65%)	32 (26.67%)		
Never	1,479 (44.29%)	70 (58.33%)		
Diabetes			<0.001	<0.001
Yes	409 (12.25%)	28 (23.33%)		
No	2,930 (87.75%)	92 (76.67%)		
Hypertension			<0.001	<0.001
Yes	1,131 (33.87%)	64 (53.33%)		
No	2,208 (66.13%)	56 (46.67%)		
Physical activity			<0.001	<0.001
No physical activity (PA = 1)	738 (22.10%)	48 (40.00%)		
Low intensity (PA = 2)	470 (14.08%)	16 (13.33%)		
Moderate intensity (PA = 3)	1,059 (31.72%)	31 (25.83%)		
High intensity (PA = 4)	1,067 (31.96%)	24 (20.00%)		
Sedentary time (min)	374.53 ± 206.73	429.12 ± 245.22	0.005	0.292
Relative total lean mass (g/kg)	632.14 ± 79.74	611.68 ± 84.00	0.006	0.02
Relative appendicular lean mass (g/kg)	274.01 ± 45.69	251.23 ± 42.98	<0.001	<0.001

* The weighted result.

Mean ± SD: *p* value was calculated by linear regression model or Kruskal-Wallis test.

%: *P* value was calculated by the chi-square test.

**Figure 3:**
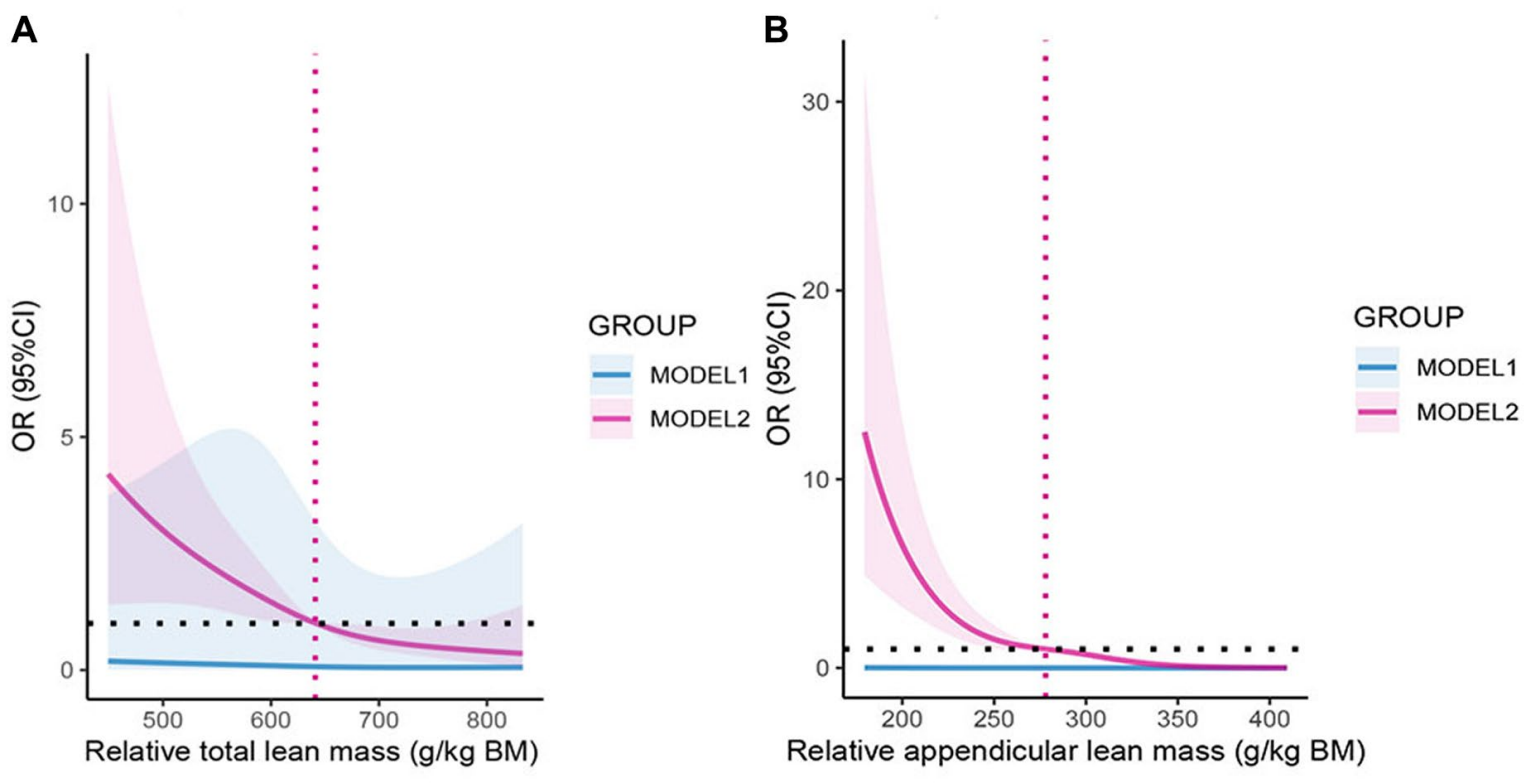
**(A)** The odds ratio of COPD with relative total lean mass, NHANES 2013–2018. **(B)** The odds ratio of COPD with relative appendicular lean mass, NHANES 2013–2018. OR: Odds ratio; CI: Confidence interval. MODEL1: No variables were adjusted; MODEL2: Adjusted for age, gender, race, educational level, PIR, BMI, smoking, alcohol drinking, physical activity, sedentary time, diabetes, and hypertension. The shaded part represents the 95% confidence interval.

Figure 3 shows the relationship between two kinds of lean mass and COPD through restricted cubic spline (RCS) model analysis. After adjustment for age, gender, race, educational level, PIR, BMI, smoking, alcohol drinking, physical activity, sedentary time, diabetes, and hypertension, we found a relative total lean mass and relative appendicular lean mass both were negatively associated with COPD risk (*p* < 0.05), and the nonlinear tests were not significant. This result further supported our hypothesis, and we then performed multiple logistic regression to explore the relationship in detail.

In a follow-up analysis, participants were grouped according to quintiles of relative lean mass (Q1: 0–20%; Q2: 20–40%; Q3: 40–60%; Q4: 60–80%; Q4: 80–100%), and the lowest mass group (Q1) was used as a reference for the analysis (Table 2). In addition, combining RCS analysis and logistic regression analysis of quintiles, we rationalized the population into three groups of the high, medium, and low lean mass to further analyze the risk level of COPD in the population for interpretation purposes. In all models (Table 3), relative appendicular lean mass was significantly inversely associated with the risk of COPD in all study populations (Model 3: OR = 0.985, 95%CI: 0.975–0.995). Moreover, the OR that compared high mass (317.0–408.5) g/kg to low mass (155.3–254.7) g/kg of relative appendicular lean was 0.214 (95% CI: 0.060–0.767), which we adjusted all the variables in Model 3. We also found that their correlation persisted in the logistic regression of Model 1 and Model 2 (Model 1: OR = 0.175, 95%CI: 0.08–0.376; Model 2: OR = 0.200, 95%CI: 0.060–0.663), However, relative total lean mass was not found to be solidly associated with COPD. The results of the constructed quintile model also remained robust in all variable adjustments, but all results could not effectively account for causality, so we applied Mendelian randomization for the next step of the study.

**Table 2:** Odds ratios (95% confidence intervals) of COPD across quintiles of relative total lean mass and relative appendicular lean mass, NHANES 2013–2018.

	Model 1	Model 2	Model 3
Relative total lean mass			
Q1 (432.7–552.5) g/kg	Ref.	Ref.	Ref.
Q2 (552.5–602.5) g/kg	0.871 (0.468,1.622)	0.940 (0.498,1.775)	0.965 (0.451,2.068)
Q3 (602.5–659.4) g/kg	0.651 (0.331,1.280)	0.839 (0.375,1.878)	0.710 (0.259,1.945)
Q4 (659.4–708.0) g/kg	0.355 (0.147,0.855)*	0.546 (0.150,1.989)	0.453 (0.142,1.442)
Q5 (708.0–838.5) g/kg	0.678 (0.341,1.346)	1.217 (0.308,4.801)	0.589 (0.156,2.224)
Relative appendicular lean mass			
Q1 (155.3–229.7) g/kg	Ref.	Ref.	Ref.
Q2 (229.7–254.7) g/kg	0.719 (0.378,1.368)	0.735 (0.383,1.411)	0.834 (0.376,1.851)
Q3 (254.7–286.5) g/kg	0.576 (0.299,1.112)	0.579 (0.235,1.431)	0.679 (0.263,1.755)
Q4 (286.5–317.0) g/kg	0.498 (0.255,0.975)*	0.505 (0.179,1.429)	0.661 (0.214,2.045)
Q5 (317.0–408.5) g/kg	0.150 (0.068,0.332)**	0.161 (0.047,0.558)**	0.183 (0.041,0.814)*

Ref: reference.

Model 1 non-adjusted model.

Model 2 adjusted for age, gender, and race.

Model 3 adjusted for age, gender, race, educational level, family income, BMI, smoking, alcohol drinking, diabetes, and hypertension.

^*^p < 0.05.

^**^p < 0.01.

**Table 3:** Odds ratios (95% confidence intervals) of COPD across three mass groups of relative total lean mass and relative appendicular lean mass, NHANES 2013–2018.

	Model 1	Model 2	Model 3
Relative total lean mass			
Overall situation	0.997(0.994,1.000)*	1.000(0.994,1.006)	0.996(0.991,1.002)
Low mass (432.7–602.5) g/kg	Ref.	Ref.	Ref.
Medium mass (254.7–317.0) g/kg	0.530(0.308,0.912)*	0.767(0.343,1.712)	0.632(0.283,1.409)
High mass (708.0–838.5) g/kg	0.725(0.385,1.365)	1.404(0.398,4.949)	0.666(0.212,2.092)
Relative appendicular lean mass			
Overall situation	0.987(0.982,0.993)**	0.984(0.973,0.995)**	0.985(0.975,0.995)**
Low mass (155.3–254.7) g/kg	Ref.	Ref.	Ref.
Medium mass (254.7–317.0) g/kg	0.625(0.381,1.026)	0.656(0.284,1.515)	0.760(0.362,1.597)
High mass (317.0–408.5) g/kg	0.175(0.081,0.376)**	0.200(0.060,0.663)**	0.214(0.060,0.767)*

Ref: reference.

Model 1 non-adjusted model.

Model 2 adjusted for age, gender, and race.

Model 3 adjusted for age, gender, race, educational level, PIR, BMI, smoking, alcohol drinking, physical activity, sedentary time, diabetes, and hypertension.

*p < 0.05.

**p < 0.01.

### 3.2. The results of bidirectional MR analysis

Three GWAS with large-scale populations were included in the Mendelian randomization analysis. In the forward two-sample MR Analysis, 201 or 97 SNPs related to appendicular lean mass were selected (through linkage disequilibrium (LD) analysis, *r*^2^ = 0.001, kb = 10,000; Supplementary Tables S1, S2), which were derived from two MR analyzes with COPD or Lung function (FEV_1_/FVC) outcomes, respectively. In addition, confounders were excluded to achieve no significant correlation between instrumental and outcome variables. If the F-statistic of an SNP is less than 10, it indicates the presence of a weak instrumental variable. Therefore, we calculated and removed the weak instrumental variables and then performed MR analysis again to examine the robustness of the results. SNPs after weak instrumental variable exclusion (Supplementary Tables S3, S4).

**Figure 4:**
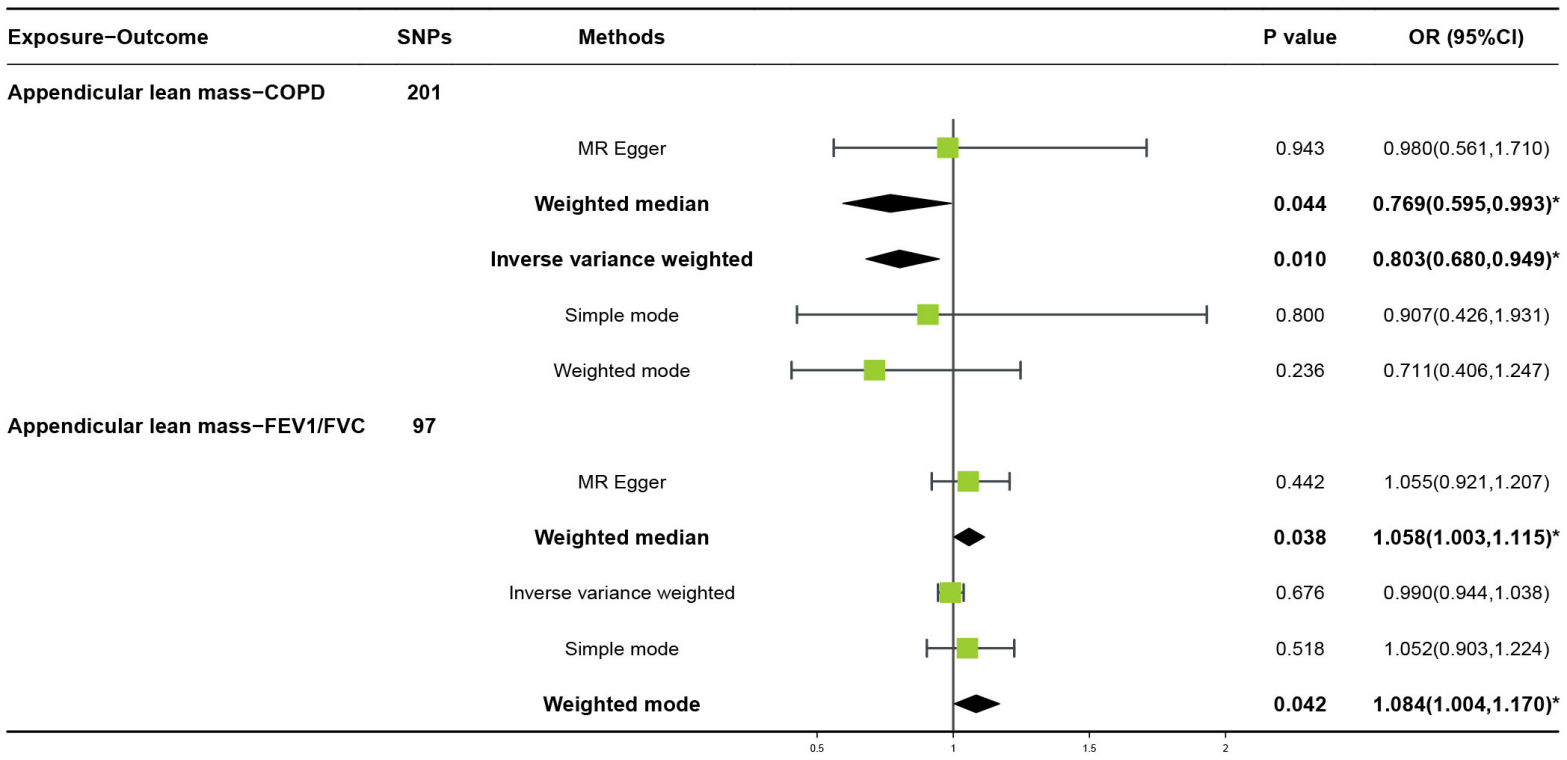
Forest plots of MR study using appendicular lean mass with COPD or FEV_1_/FVC.

**Figure 5:**
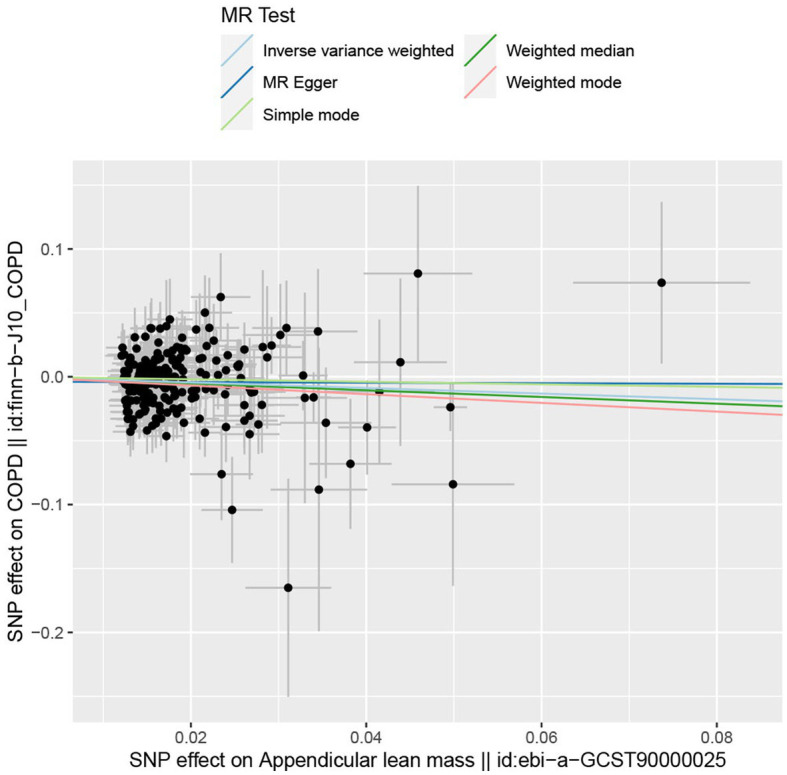
The scatter plot for MR analyzes of causal associations between each appendicular lean mass SNP and COPD.

**Figure 6:**
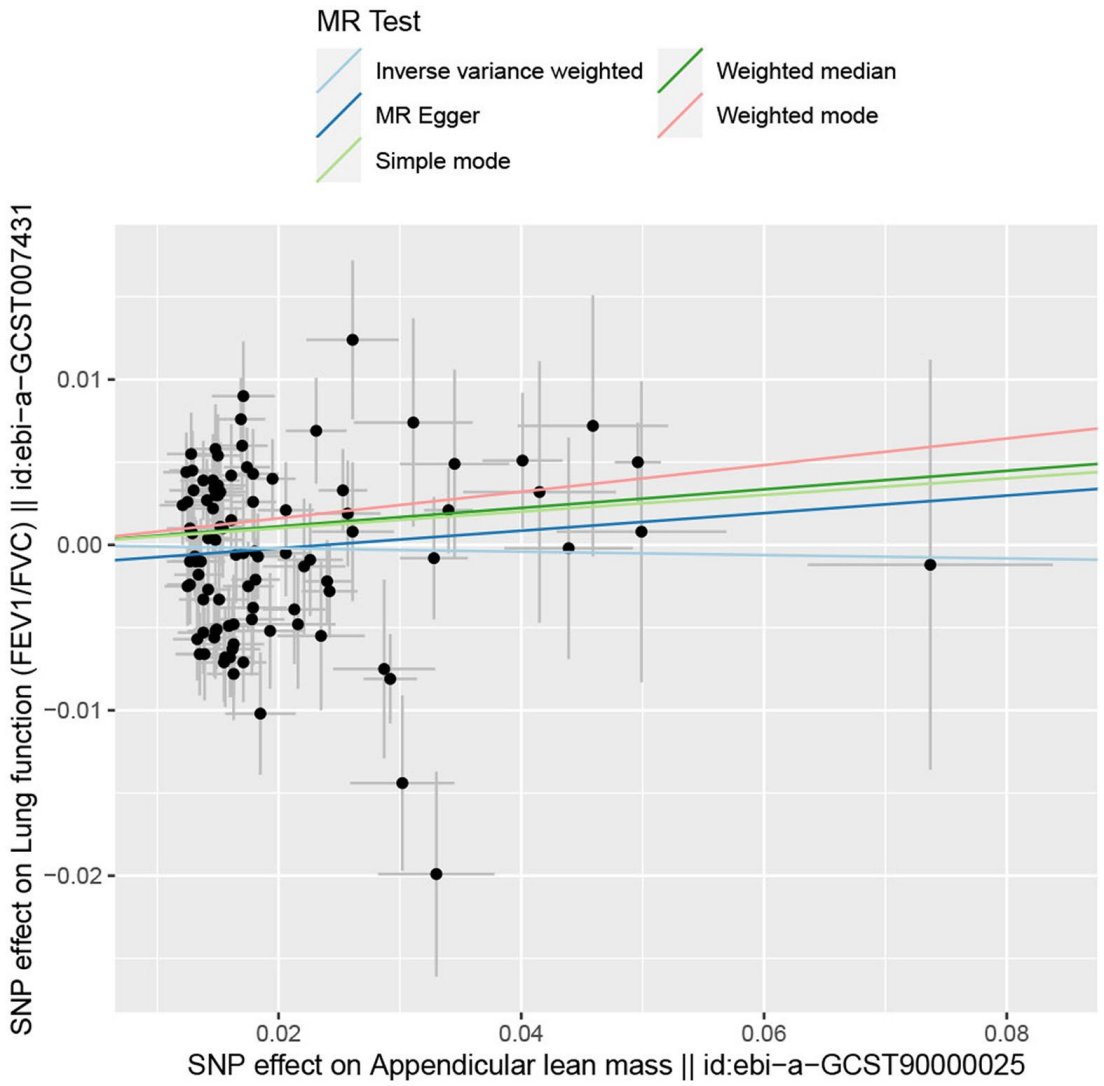
The scatter plot for MR analyzes of causal associations between each appendicular lean mass SNP and FEV_1_/FVC.

In the forward MR analysis, the IVW found an association between appendicular lean mass and risk of COPD (OR = 0.803; 95%CI: 0.680–0.949; *p* = 0.010; Figure 4). A similar trend of results was observed in the other models (Figure 5) and there was no heterogeneity (Q = 179.146; *p* = 0.853). Visualization of heterogeneity results is shown in the funnel plot (Supplementary Figure S1). The pleiotropy test did not identify pleiotropy for IVs (*p* = 0.465). After excluding instrumental variables, the IVW model findings remained significant (OR = 0.800; 95%CI: 0.669–0.956; *p* = 0.014; Supplementary Figure S2). Sensitivity analysis of the leave-one-out method revealed that the combined effect of removing any individual SNP remained almost constant, indicating that the results were plausible (Supplementary Figure S3; Supplementary Table S5) and forest plots for each SNP in the appendicular lean mass-COPD analysis were shown in Supplementary Figure S4 and Supplementary Table S6. In contrast, although the causal effect of appendicular lean mass on lung function (FEV1/FVC) was not significant in the IVW model (*p* = 0.676), the two prominent models exhibited a trend toward a positive effect on lung function as well (Figure 6).

In the reverse two-sample MR analysis, 4 or 166 SNPs related to COPD or lung function (FEV1/FVC) were selected (through LD analysis, *r*^2^ = 0.001, kb = 10,000; Supplementary Tables S7, S8), which the outcome factor was appendicular lean mass. The results of the IVW model, with or without the removal of the weak instrumental variables, suggested that there was no causal relationship between COPD (*p* = 0.384) or lung function (*p* = 0.716) on the appendicular lean mass (Supplementary Figure S5).

## 4. Discussion

This study used epidemiological cross-sectional analysis and two-sample bidirectional MR analysis to explore the causality between lean mass and COPD risk. Our results support the association between appendicular lean mass and COPD risk as well as further confirm a possible forward causality in findings.

Body composition has been linked to physical performance (38). In particular, the relationship between BMI and COPD has been extensively studied and confirmed (39). In a large cohort study involving 12,396 multi-country subjects, Grigsby et al. discovered that people with lower BMI were at higher risk of COPD and lower lung function compared to those with higher BMI. Even after excluding patients with COPD, the association between being underweight and lung function remained negative, indicating that being underweight may also lead to deterioration of lung function (40). In addition, Trethewey et al. revealed that lower BMI was related to a precipitous decrease in FEV_1_/FVC% (41). These studies demonstrate that BMI may precede and play an important role in the progression of COPD pulmonary pathophysiology. However, few have studied and confirmed such associations with changes in COPD and measures of lean mass. A study by Behrens et al. concerning 113,279 participants showed that the incidence of COPD was high in both severely obese and underweight participants, but after adjustment for waist circumference which suggested low muscle levels, only underweight remained positively associated with COPD (8). Similarly, a cross-sectional study from Peru reported that COPD is associated with being underweight and having less lean mass, which differs from other chronic respiratory diseases (CRDs) (42). These studies may have implications for the importance of lean mass on the risk of COPD.

Lean mass, like BMI, has been studied not infrequently in COPD patients, but lean mass appears to be relatively understudied in the inclusion of non-COPD. Both relative skeletal muscle mass index (RSMI) and BMI decreased as COPD worsened (43). Related studies indicated that low BMI was not only a systemic consequence of COPD but also a major risk factor for COPD development, which increases the possibility that early intervention in patients with low BMI may reduce the risk of COPD (7). Nevertheless, based on previous studies, it is uncertain whether lean mass is an important risk factor. A remarkably high prevalence of low body lean mass was observed in COPD patients, disease severity and functional outcomes were associated with adverse muscle outcomes, as well as a low lean mass was associated with FEV_1_% < 50% (44, 45). Being Underweight has long been associated not only with COPD but also with higher mortality in COPD patients (46). Regardless of fat mass, fat-free mass is an independent predictor of mortality (47). Muscle loss contributes to higher morbidity and mortality in COPD (48). Furthermore, we found evidence that cross-supported the findings of this paper, predicting that lean mass may be a risk factor for COPD. A cross-sectional study of 452 patients with COPD and 459 patients without COPD showed that lean mass reduction in COPD patients was correlated with the risk of emphysema (49). In addition, a logistic regression model involving airflow obstruction in another study showed that odds ratios for low lung density with lean mass index in men (OR = 0.85) and women (OR = 0.74) were also significant (41).

The association between low lean mass and a high incidence of COPD can be interpreted by several possible factors. One possible explanation for the association in adults is the discovery that defective cell-mediated immunity and circulating T-lymphocyte counts resulting from protein-energy malnutrition can lead to increased susceptibility to infection (39), which is especially important for COPD, as minor respiratory infections may severely impair lung function. Numerous studies have shown the possible use of nutritional interventions for patients with COPD. Increasing caloric consumption through nutritional interventions can gain weight and muscle strength, improving the QOL in COPD patients (50). Besides, all patients showed significant improvement in fat-free mass, and lean body mass compared to baseline after the nutritional intervention (51). The ketogenic diet (KD) is also recognized as a nutritional intervention with a low-carbohydrate but high-fat diet, providing supplements and metabolic flexibilities in skeletal muscles for enhancing exercise performances (52), which replaces glucose sugar with ketone bodies and is effective in several diseases, including metabolic disorders and skeletal muscle atrophy (53). Antioxidant and anti-inflammatory characteristics allow KD to enhance endurance, strength, and exercise performance as well as prevent exercise-induced muscle and organ weakness (54). These all seem to favor COPD prevention from a muscular perspective, but the effects and mechanisms of its role are unknown and deserve to be studied in the future. However, nutrition interventions alone did not seem to reverse fat-free mass reduction (55). Popular approaches to alleviate COPD-related sarcopenia currently include pulmonary rehabilitation and nutritional interventions (56), which suggest that both interventions together in underweight patients are associated with improvements in muscle strength, exercise performance, and QOL. A meta-analysis related to nutritional support also found significant improvements in grip strength and weight gain of more than 2 kg in patients, while it led to enhanced exercise programs (57).

A study demonstrating an association between systemic inflammatory markers (IL-8, CRP, and TNF-α) and upper extremity muscle strength showed a negative effect of systemic inflammation on muscle strength in individuals with stable COPD (58). Systemic inflammation caused by elevated TNF-α production and higher resting energy expenditure due to metabolic and mechanical inefficiencies was associated with underweight and reduced fat-free mass. Systemic inflammatory mediators enhance protein hydrolysis and inhibit protein synthesis, leading to loss of muscle mass with adverse effects on skeletal muscle (59). Experimental studies have shown that elevated blood levels of TNF-α promote muscle atrophy by enhancing the activity of the ubiquitin-proteasome pathway or by inducing apoptosis (60). It may also affect muscles by directly impairing contractile function (58, 61). Furthermore, skeletal muscle is an essential immunomodulatory organ that produces a series of proteins called myokines that have anti-inflammatory and immunoprotective effects, which may provide explanatory directions for muscle maintenance and protection against COPD risk in the population (62). Notably, physical activity (PA) and exercise were proven countermeasures against muscle aging and have been shown to reduce the decline in lean mass, strength, and regenerative capacity, and also to slow or prevent impaired muscle metabolism (63, 64). Lack of or reduced levels of PA are part of the underlying mechanisms of sarcopenia, and PA can be considered an important factor in reversing or altering the development of this condition. Undoubtedly, aerobic and resistance exercises are the most vital methods for the prevention and treatment of sarcopenia (65). Interestingly, A certain amount of PA per week was associated with a reduced risk of COPD, which may include a reduction in oxidative stress and chronic inflammation caused by PA (66, 67). Habitual PA levels decline with age and have a significant impact on muscle mass and function, improved PA may contribute to the prevention of COPD by maintaining lean mass (8, 64). We were aware of the lack of muscle regeneration potential in patients with sarcopenic COPD (68), and resistance training (RT) was an important management of muscle dysfunction in COPD (69–71). Evidence supported that exercise in patients with COPD had beneficial effects on the strength and exercise capacity of peripheral skeletal muscles (72). Additionally, participation in muscle-strengthening activities was associated with higher survival rates in patients with COPD (73). Therefore, resistance training may also be effective in the prevention of COPD by acting on skeletal muscle through resistance training to maintain muscle mass and muscle function. Also beyond preventive interventions, identification is equally important. The findings of this study have implications for future COPD prevention and screening with a focus on lean mass that could be considered for inclusion with the aid of Dual-energy X-ray (DXA), Bioelectrical impedance analysis (BIA), or Ultrasonography (74).

There was no strong evidence for the reverse causal relationship between appendicular lean mass on COPD or lung function (FEV_1_/FVC) in the MR study in this paper. It is possible that the significant co-morbid sarcopenia in COPD is not directly due to pulmonary function aspects, but may be the consequence of dyspnea, decreased exercise tolerance, lack of physical activity (75, 76), and systemic chronic systemic inflammation (77) in COPD patients resulting in reduced lean mass, which these factors are major risk factors for skeletal muscle dysfunction (SMD) in patients with COPD.

The main strengths of this study include the large cross-sectional study based on NHANES and the two-sample bidirectional MR analysis study whose findings corroborate each other to a high degree of confidence. Cross-sectional studies can explore the relationship between lean mass and COPD risk from epidemiology based on DEXA examination, and MR overcomes the traditional effects of numerous confounding factors and reverse causality bias in epidemiological cross-sectional studies, exploring the causal relationships involved. The second advantage is the further refinement of the effect of body composition on COPD risk, compared to previous studies we found that the effect of appendicular lean mass contributes to COPD prevention and detection, which may also help the future of COPD identification. However, there are some potential limitations to this study. First, there may be a self-reported recall bias for COPD diagnosis in cross-sectional studies. Second, the results may be biased and cannot be extended to other populations, as the data are limited to the US and European populations. Because of the shortcomings limited to cross-sectional studies and Mendelian randomization, further corroboration from higher levels of evidence-based studies such as RCTs and longitudinal studies will be needed.

## 5. Conclusion

Our study highlighted a causal association between appendicular lean mass and COPD risk, higher lean mass may be a protective factor for COPD, and people with low appendicular lean mass are at high risk for COPD. In the future, it will be necessary to consider the possible effects of resistance training, physical activity intervention, and nutritional supplement on the prevention of COPD by maintaining muscle mass.

## Data availability statement

Publicly available datasets were analyzed in this study. This data can be found at: https://wwwn.cdc.gov/nchs/nhanes/Default.aspx.

## Ethics statement

Ethical review and approval was not required for this study since it involves secondary data analysis from the NHANES. Informed consent was obtained from all individual participants included in the study.

## Author contributions

CF and HY contributed to the conception and design of the study. CF organized the data and performed the statistical analysis and wrote the first draft of the manuscript. HY wrote sections of the manuscript. All authors contributed to the manuscript revision, read, and approved the submitted version.

## Funding

This research was funded by Postgraduate Research and Practice Innovation Program of Jiangsu Province in 2023, grant number KYCX23_0641; the Excellent Master’s Thesis Selection and Cultivation Program of Hohai University in 2022, grant number 422003506; and Project of Hohai University Discipline Action Plan, grant number 1013-418246.

## Conflict of interest

The authors declare that the research was conducted in the absence of any commercial or financial relationships that could be construed as a potential conflict of interest.

## Publisher’s note

All claims expressed in this article are solely those of the authors and do not necessarily represent those of their affiliated organizations, or those of the publisher, the editors and the reviewers. Any product that may be evaluated in this article, or claim that may be made by its manufacturer, is not guaranteed or endorsed by the publisher.

## Footnotes

^1^https://wwwn.cdc.gov/nchs/nhanes/Default.aspx

^2^https://www.ukbiobank.ac.uk/

^3^https://www.finngen.fi/en/

^4^https://gwas.mrcieu.ac.uk/

^5^www.r-project.org
