# Supplementary material for: Association between appendicular lean mass and chronic obstructive pulmonary disease: epidemiological cross-sectional study and bidirectional Mendelian randomization analysis
Source: Front Nutr. 2023 Jun 29;10:1159949. doi: 10.3389/fnut.2023.1159949 (PMC10338881; doi:10.3389/fnut.2023.1159949)
Supplement: Supplementary file 1 [file Data_Sheet_1.PDF]

Supplementary Material

Association between appendicular lean mass and chronic obstructive pulmonary disease: Epidemiological cross-sectional study and Bidirectional mendelian randomization analysis

Chengjie Fu, Qifei Wang, Hongchang Yang*

*** Correspondence:** yanghongchang@hhu.edu.cn

1 Supplementary Figures and Tables

1.1 Supplementary Figures

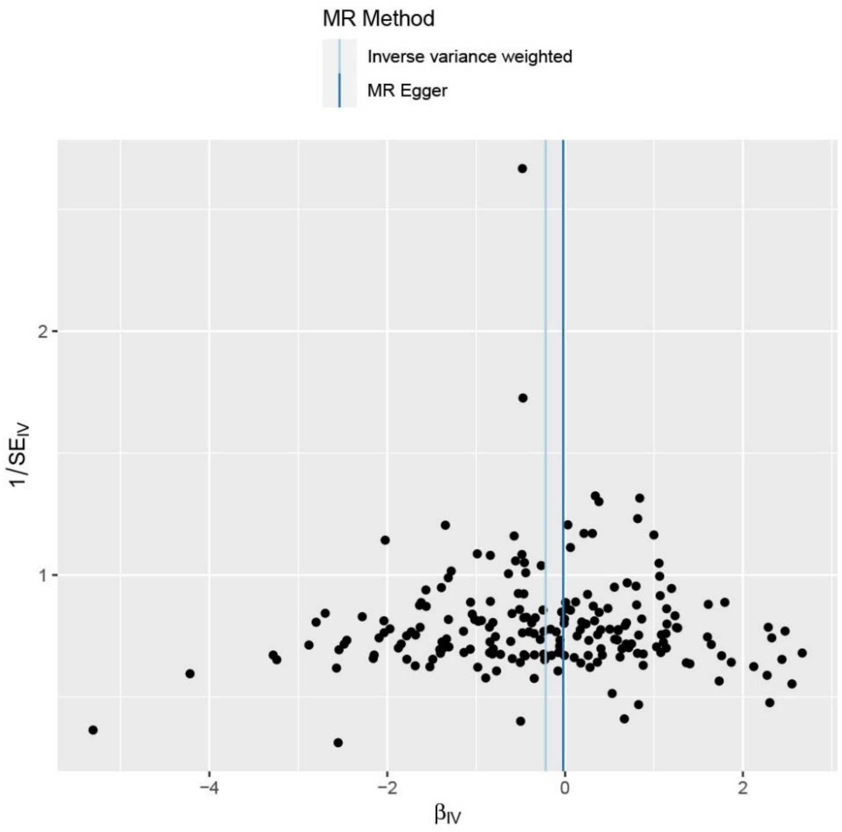

Supplementary Figure 1. The funnel plot for MR analyses of forward causal associations between each appendicular lean mass SNP and COPD.

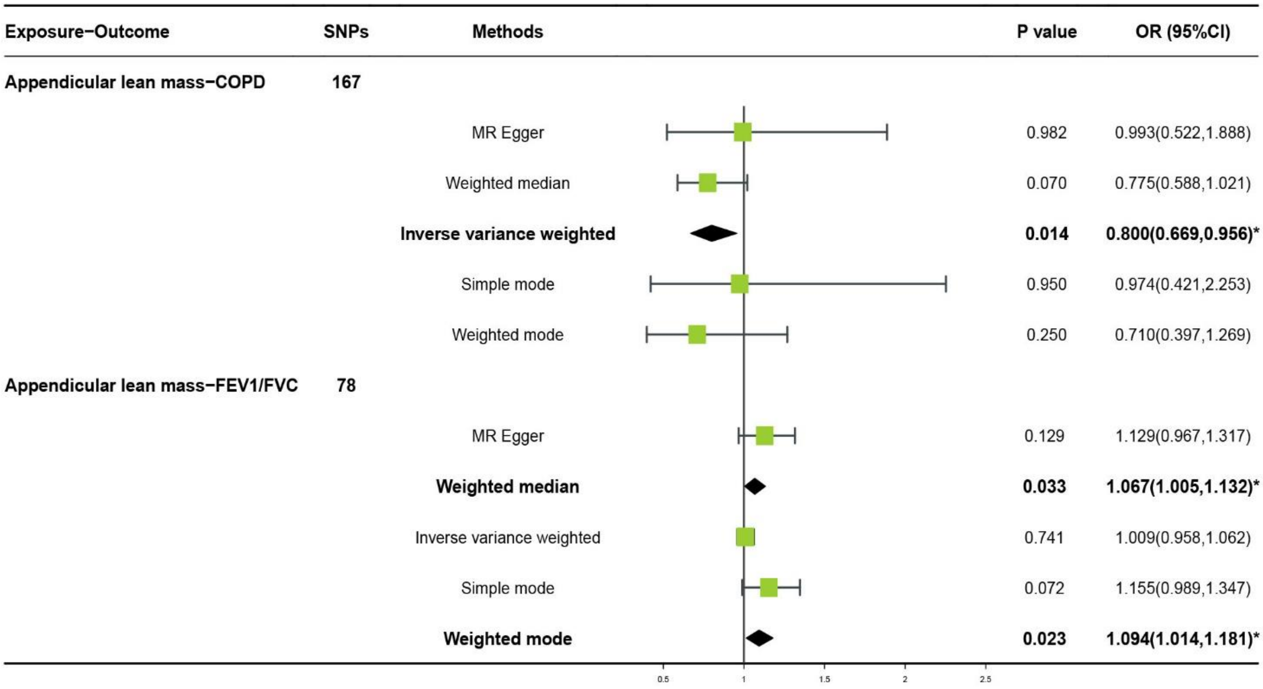

Supplementary Figure 2. Forest plots of MR study using appendicular lean mass with COPD or FEV_1/FVC (weak IVs were excluded).

Supplementary Figure 3. Leave-one-out sensitivity analysis for COPD using SNP associated appendicular lean mass.

Supplementary Figure 4. The forest plot for MR analyses of causal associations between each appendicular lean mass SNP and COPD.

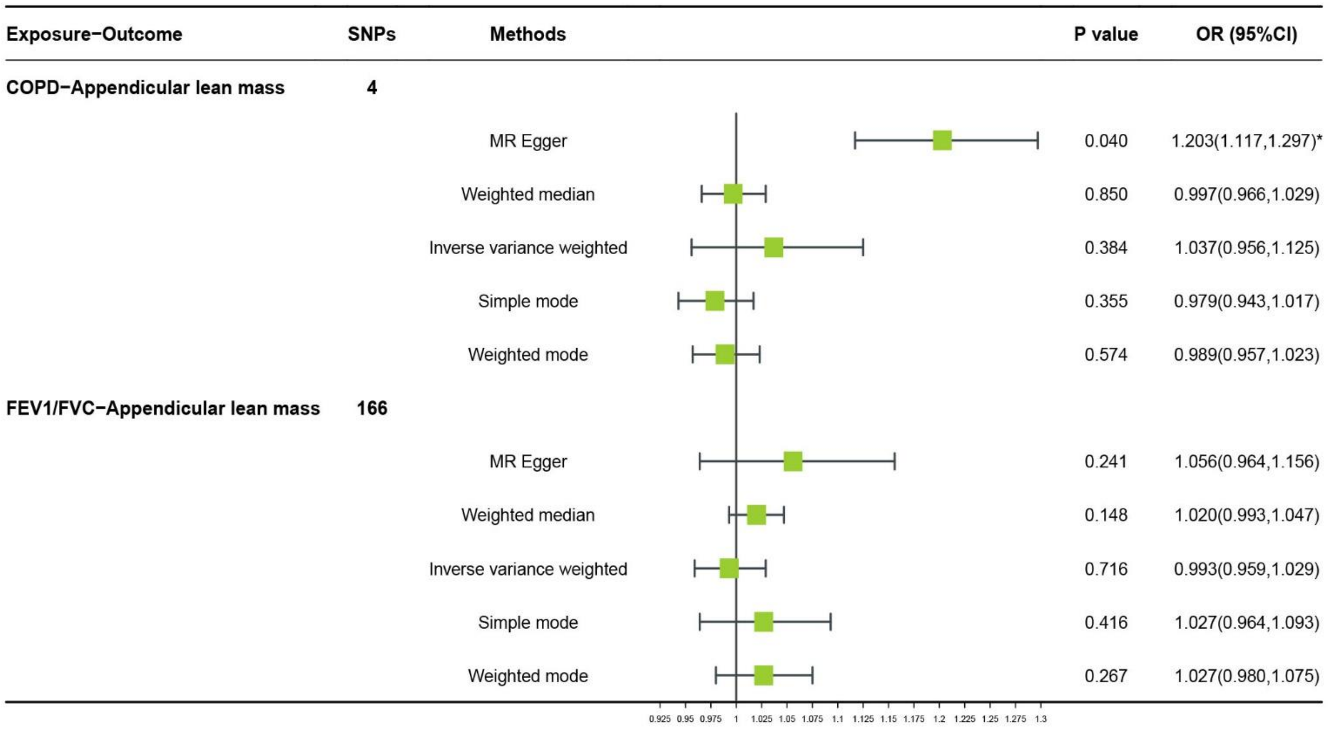

Supplementary Figure 5. Forest plots of MR study using COPD or FEV_1/FVC with appendicular lean mass.

1.2 Supplementary Tables

Supplementary Table 1. Characteristics of SNPs associated with appendicular lean mass and COPD.

Supplementary Table 2. Characteristics of SNPs associated with appendicular lean mass and FEV_1/FVC.

Supplementary Table 3. Characteristics of SNPs associated with appendicular lean mass and COPD (R^2 and F values were calculated & weak IVs were excluded).

Supplementary Table 4. Characteristics of SNPs associated with appendicular lean mass and FEV_1/FVC (R^2 and F values were calculated & weak IVs were excluded).

Supplementary Table 5. Leave-one-out sensitivity analysis for COPD using SNP associated appendicular lean mass.

Supplementary Table 6. The MR analyses of causal associations between each appendicular lean mass SNP and COPD.

Supplementary Table 7. Characteristics of SNPs associated with COPD.

Supplementary Table 8. Characteristics of SNPs associated with FEV_1/FVC.
