# Supplementary figures and images for: Association between appendicular lean mass and chronic obstructive pulmonary disease: epidemiological cross-sectional study and bidirectional Mendelian randomization analysis
Source: Front Nutr. 2023 Jun 29;10:1159949. doi: 10.3389/fnut.2023.1159949 (PMC10338881; doi:10.3389/fnut.2023.1159949)

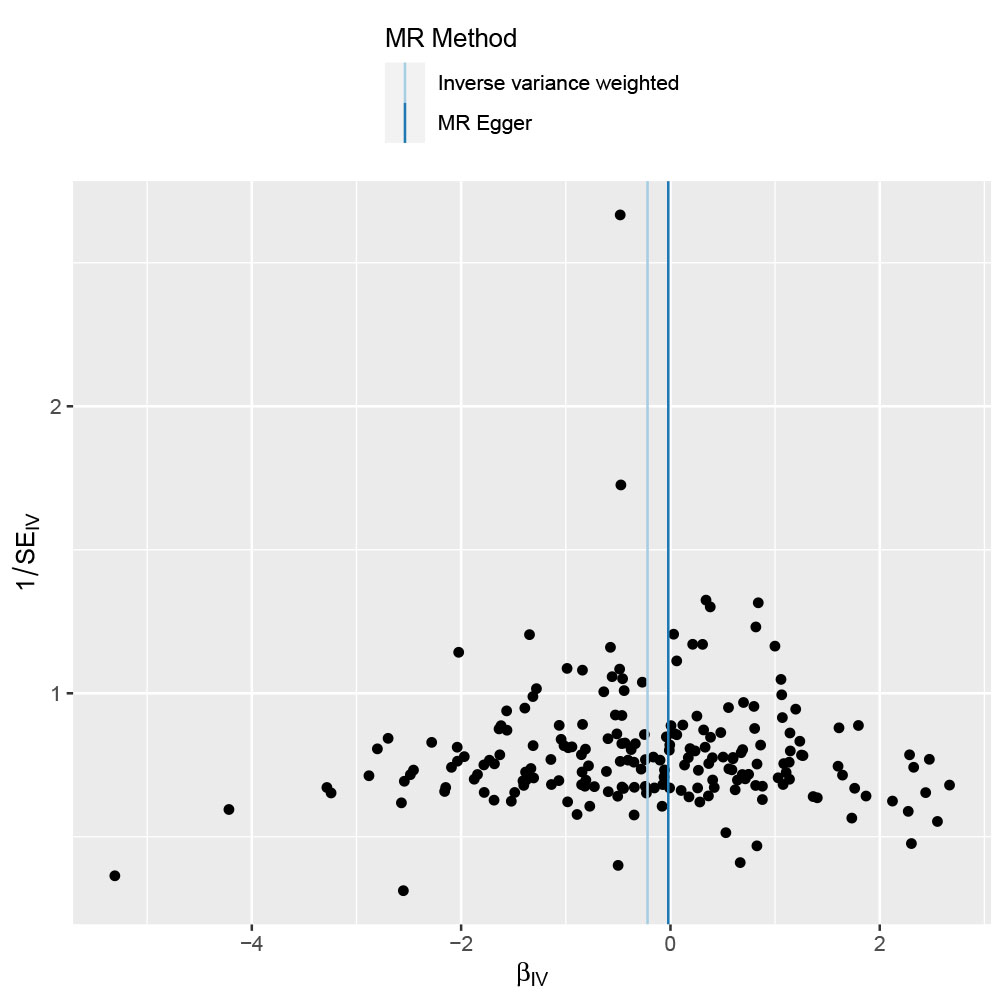

Supplement: Supplementary file 2 [file Data_Sheet_2.ZIP › Supplementary Figure 1.jpg]

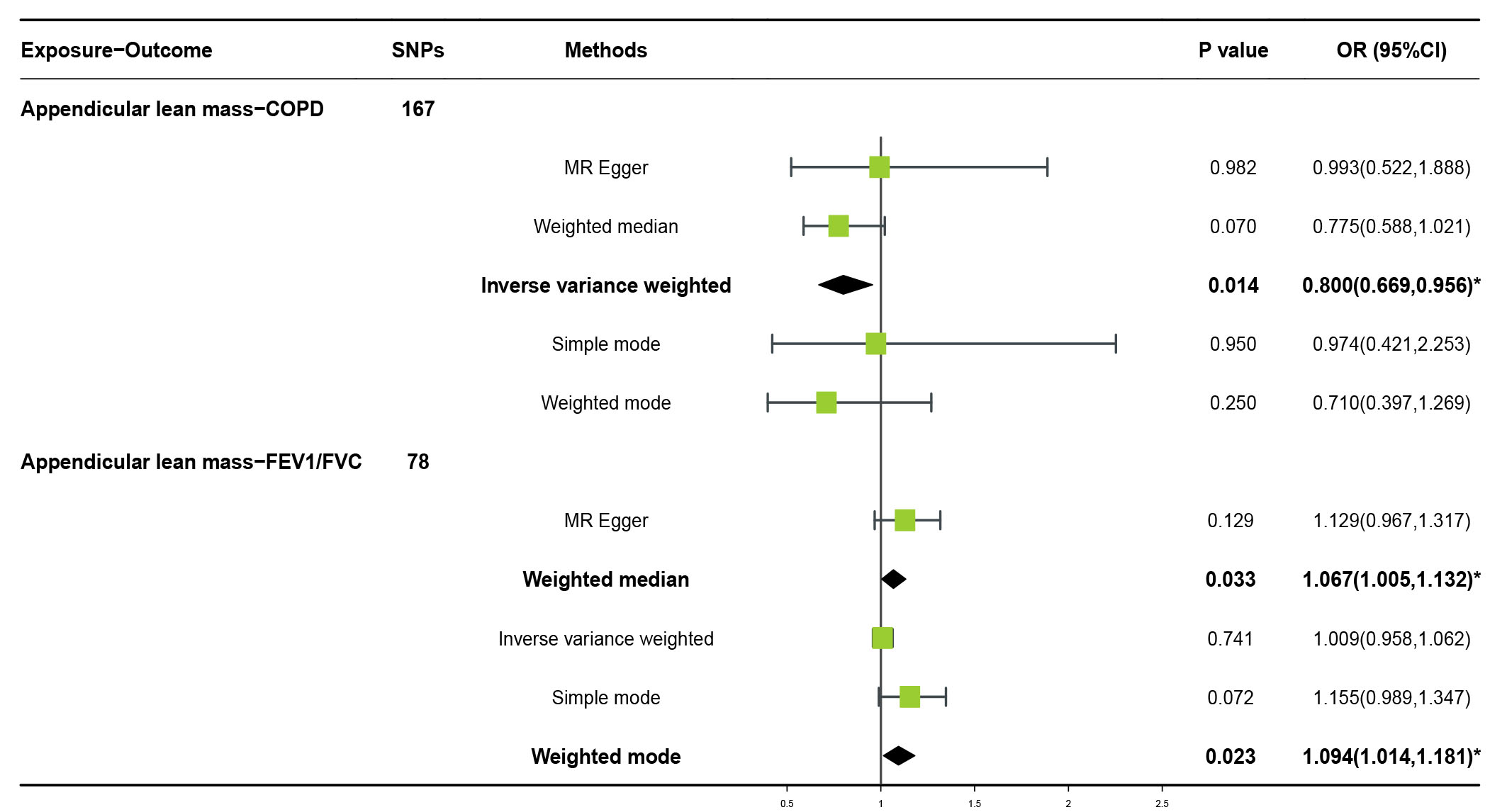

Supplement: Supplementary file 2 [file Data_Sheet_2.ZIP › Supplementary Figure 2.jpg]

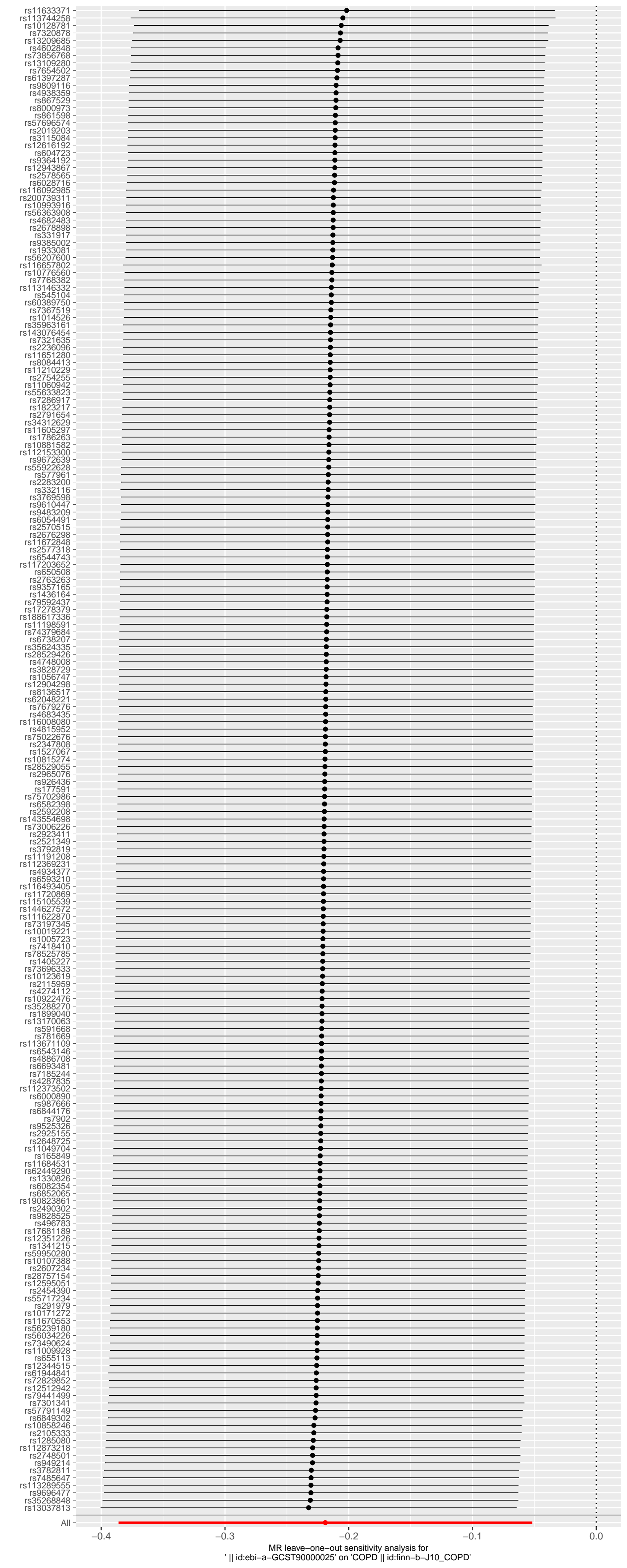

Supplement: Supplementary file 2 [file Data_Sheet_2.ZIP › Supplementary Figure 3.pdf]

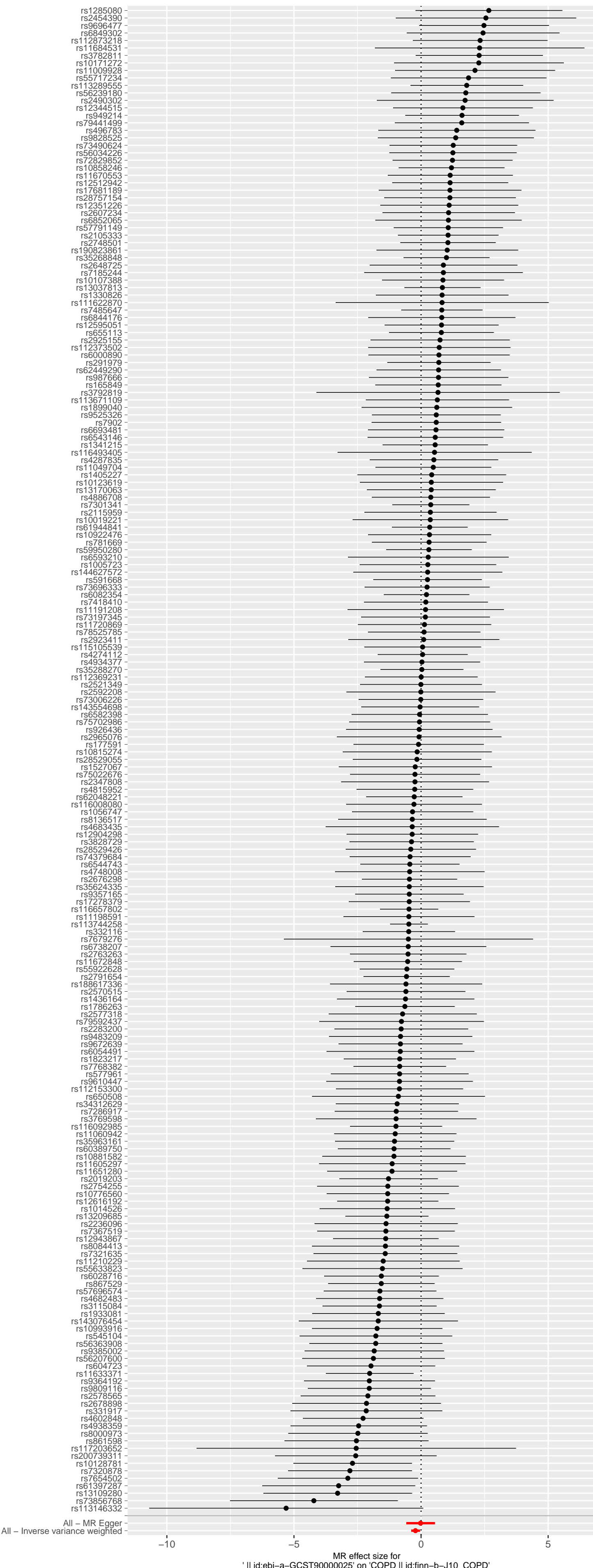

Supplement: Supplementary file 2 [file Data_Sheet_2.ZIP › Supplementary Figure 4.pdf]

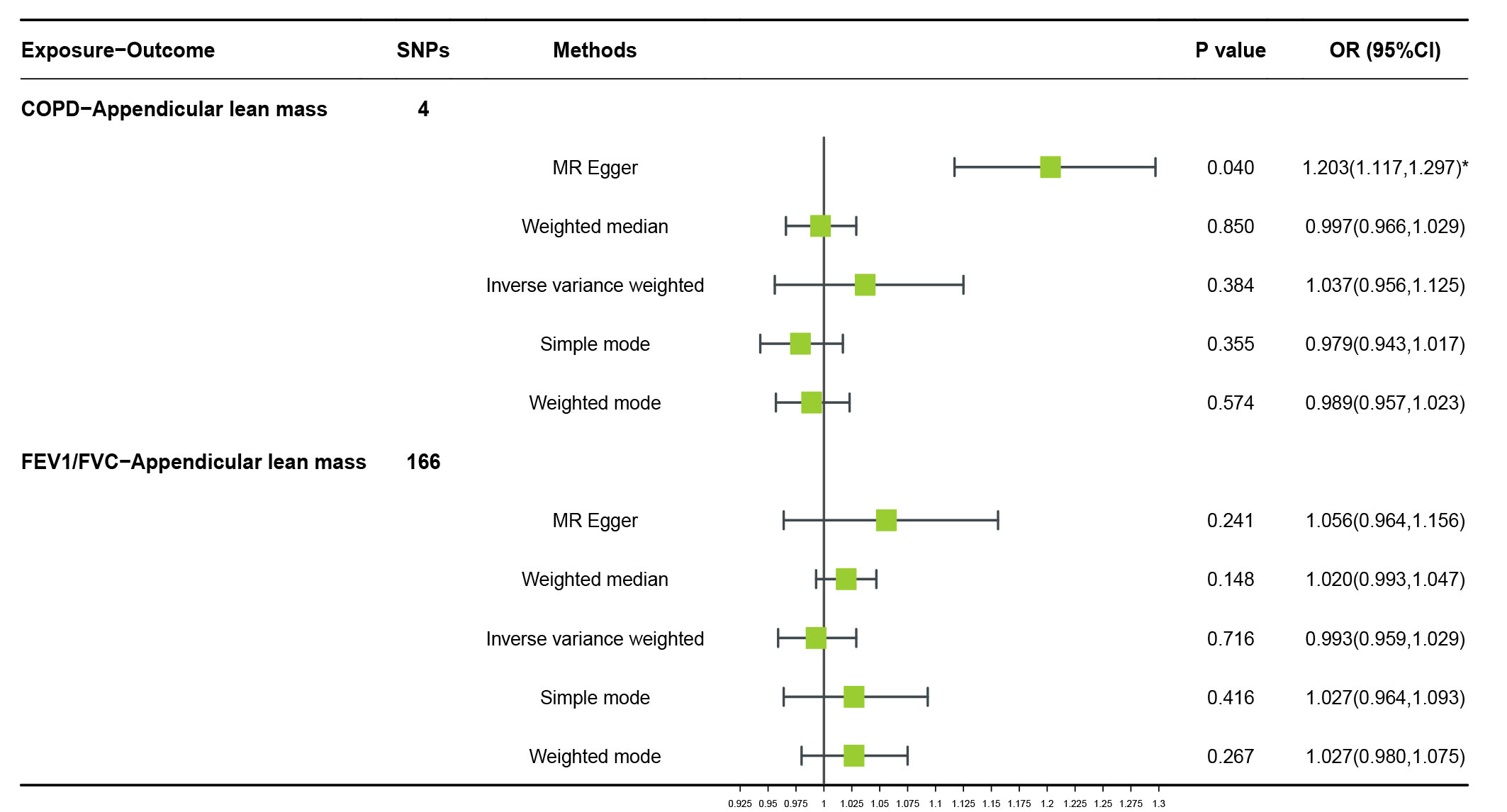

Supplement: Supplementary file 2 [file Data_Sheet_2.ZIP › Supplementary Figure 5.jpg]
